# Synthesis, Molecular Docking Screening and Anti-Proliferative Potency Evaluation of Some New Imidazo[2,1-*b*]Thiazole Linked Thiadiazole Conjugates

**DOI:** 10.3390/molecules25214997

**Published:** 2020-10-28

**Authors:** Huda R. M. Rashdan, Aboubakr H. Abdelmonsef, Ihsan A. Shehadi, Sobhi M. Gomha, Abdel Mohsen M. Soliman, Huda K. Mahmoud

**Affiliations:** 1 Chemistry of Natural and Microbial Products Department, Pharmaceutical and Drug Industries Research Division, National Research Centre, Dokki, Cairo 12622, Egypt; 2 Chemistry Department, Faculty of Science, South Valley University, Qena 83523, Egypt; aboubakr.ahmed@sci.svu.edu.eg; 3 Chemistry Department, Faculty of Science, University of Sharjah, Sharjah 27272, UAE; ishehadi@sharjah.ac.ae; 4 Chemistry department, Faculty of Science, Cairo University, Giza 12613, Egypt; s.m.gomha@gmail.com (S.M.G.); H-kamel2011@cu.edu.eg (H.K.M.); 5 Department of Chemistry, Faculty of Science, Islamic University in Almadinah Almonawara, Almadinah Almonawara 42351, Saudi Arabia; 6 Therapeutic Chemistry Department, National Research Centre, Dokki, Cairo 12622, Egypt; amsolimannrc@gmail.com

**Keywords:** imidazo[2,1-*b*]thiazole, hydrazonoylhalides, thiadiazoles, molecular docking studies, anti-proliferative activity

## Abstract

Background: Imidazo[2,1-*b*]thiazole scaffolds were reported to possess various pharmaceutical activities. Results: The novel compound named methyl-2-(1-(3-methyl-6-(*p*-tolyl)imidazo[2,1-*b*]thiazol-2-yl)ethylidene)hydrazine-1-carbodithioate **3** acted as a predecessor molecule for the synthesis of new thiadiazole derivatives incorporating imidazo[2,1-*b*]thiazole moiety. The reaction of **3** with the appropriate hydrazonoyl halide derivatives **4a**–**j** and **7**–**9** had produced the respective 1,3,4-thiadiazole derivatives **6a**–**j** and **10**–**12**. The chemical composition of all the newly synthesized derivatives were confirmed by their microanalytical and spectral data (FT-IR, mass spectrometry, ^1^H-NMR and ^13^C-NMR). All the produced novel compounds were screened for their anti-proliferative efficacy on hepatic cancer cell lines (HepG_2_). In addition, a computational molecular docking study was carried out to determine the ability of the synthesized thiadiazole molecules to interact with active site of the target Glypican-3 protein (GPC-3). Moreover, the physiochemical properties of the synthesized compounds were derived to determine the viability of the compounds as drug candidates for hepatic cancer. Conclusion: All the tested compounds had exhibited good anti-proliferative efficacy against hepatic cancer cell lines. In addition, the molecular docking results showed strong binding interactions of the synthesized compounds with the target GPC-3 protein with lower energy scores. Thus, such novel compounds may act as promising candidates as drugs against hepatocellular carcinoma.

## 1. Introduction

Exploring new leads for the identification of novel structures might be useful in designing less toxic selective and potent new anticancer agents and remains a major challenge for medicinal chemists. Imidazo[2,1-*b*]thiazole scaffolds have drawn a significant interest recently due to their wide spectrum of pharmaceutical activities, especially antitumor activities [1,2]. Imidazo[2,1-*b*]thiazole-guanylhydrazone derivative revealed potent anti-proliferative activities against a number of cancer cell lines and is considered as a promising lead for such therapeutic applications [3]. These fused heterocyclic compounds had shown anticancer activities via different molecular mechanisms as bytubulin polymerization and inhibiting p90 ribosomal S6 kinase 2 (RSK2) [4,5,6,7,8]. Recently, we reported that imidazo[2,1-*b*]thiazole-chalcone conjugates bind at the colchicine site and with tubulin polymerization inhibitors [1]. Nocodazole is a well-known inhibitor of tubulin polymerization, that inhibits cell proliferation and is largely used as a positive control during the biological activity studies [9,10].

Otherwise, in applied synthesis, 1,3,4-thiadiazole nucleus attracted a considerable interest, owing to their wide spectra of pharmaceutical and biochemical effect. 1,3,4-Thiadiazole derivatives possess anticancer, antioxidant, anti-inflammatory, analgesic, antifungal, anti-tuberculosis, molluscicidal, antibacterial, antileishmanial, analgesic, antihypertensive and anticonvulsant efficacy [11,12,13,14]. It has been reported that such compounds possess antitumor properties against a variety of human cancer cell lines [15,16,17]. In continuation to a previous study where we deal with the utility of hydrazonoyl halides for synthesis of various bioactive bridgehead nitrogen polyheterocycles [18,19,20,21,22,23,24,25,26,27,28,29,30,31,32], we aim herein to report a new facile synthesis of new imidazo[2,1-*b*]thiazole-linked thiadiazole conjugates, It is anticipated that the synthesized compounds will have potent pharmacological activities as anticancer agents.

On the other hand, in silico molecular docking studies play an important role in searching for an appropriate ligand that energetically and geometrically fit to the active site of the target protein [33].

Glypican-3 (GPC-3) protein is considered as a candidate therapeutic target in hepatic cancer [34]. It is a member of the glypican family of heparan sulfate (HS) proteoglycans that are attached to the cell surface by a glycosylphosphatidylinositol (GPI) anchor [35]. Earlier studies suggested that Glypican-3 is an important liver cancer specific target, as it is highly expressed in hepatocellular carcinoma but not in normal tissue [36,37,38]. It was reported that many chemotherapeutic agents which has been used for treatment of hepatic cancer have binding affinity to GPC-3 protein. As an authentic example, doxorubicin is a chemotherapeutic agent used for the treatment of liver cancers [33] and exhibited hydrogen bond interactions with TYR255, CYS401 and PRO267 respectively of GPC-3 protein.

Therefore, in this study, GPC-3 is selected as a promising target for the identification of novel potent inhibitors against hepatocellular carcinoma (HCC).

The main aim of this work is to prepare a new series of anti-hepatic cancer agents. So, a new series of imidazo[2,1-*b*]thiazole-linked thiadiazole conjugates was synthesized and then, on the basis of comparative molecular docking study to identify the inhibition potency of these compounds to GPC-3 protein, it was found that the newly synthesized compounds have potent pharmacological activities as the upregulation of Glypican-3, which associated with hepatocellular carcinoma (HCC) [18]. Finally, all the newly synthesized compounds were subjected to be tested for their anti-proliferative potency against HepG2 with their safety effects on normal cells.

## 2. Results and Discussion

### 2.1. Chemistry

**Scheme 1:**
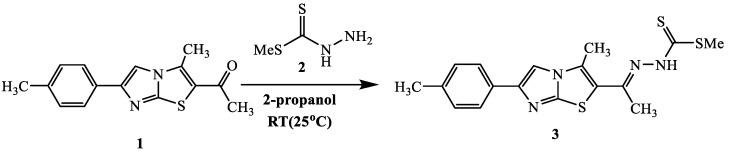
Synthesis of methyl dithioate derivative **3**.

Condensation of 1-(3-methyl-6-(*p*-tolyl)imidazo[2,1-*b*]thiazol-2-yl)ethan-1-one **1** with methyl hydrazinecarbodithioate **2** at room temperature afforded the target compound methyl-2-(1-(3-methyl-6-(*p*-tolyl)imidazo[2,1-*b*]thiazol-2-yl)ethylidene)hydrazine-1-carbodithioate **3** (Scheme 1). The structure of **3** was confirmed through the correct spectral data (IR, Mass, ^1^H-NMR) and Microanalytical data. The IR spectrum of **3** revealed the presence of the significant band for NH group and the absence of any bands for C=O. Furthermore, ^1^H-NMR spectra for **3** revealed a singlet signal at δ = 12.0 ppm for NH. Moreover, the structure of **3** was confirmed by its mass spectrum which agreed with the molecular formula (see Experimental part).

**Scheme 2:**
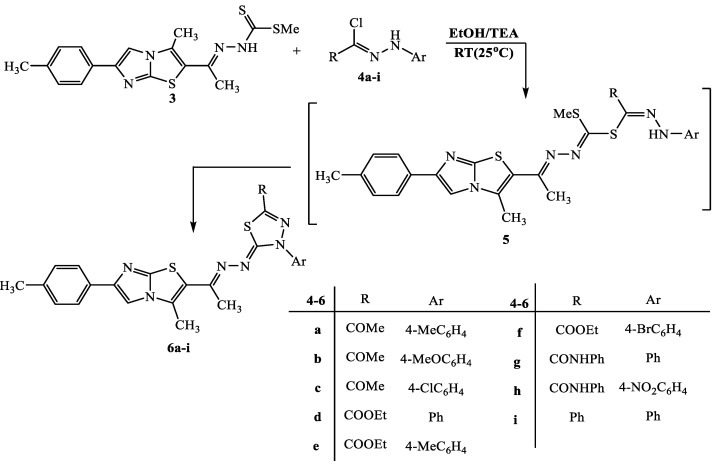
Synthesis of thiadiazole derivatives **6a**–**i**.

The chemical structure of compound **3** was confirmed further via chemical transformation. Compound **3** was reacted with a series of selected derivatives of hydrazonoyl halides in the presence of 2-3 drops of TEA to give products assigned as 2-((1-((1,3,4-thia diazolylidene)hydrazono)ethyl)-3-methyl-6-phenylimidazo[2,1-*b*]thiazoles **6a**–**i** (Scheme 2). The identity of compounds **6a**–**i**, was concluded based on the corresponding spectral and microanalytical data. The IR spectra of **6b**, for example, showed a strong absorption band at 1671 cm^−1^, which suggested the presence of carbonyl (-C=O), and the disappearance of absorption band of the amine group (–NH). Moreover, ^1^H-NMR spectrum of **6b** had revealed signals at δ = 2.31, 2.34, 2.49 and 2.54 ppm for protons of the four methyl groups, plus a new characteristic singlet signal at 3.88 ppm which was assigned for protons of methoxy group (–OMe), and singlet signal at 7.28 ppm for imidazole proton. Hence, the formation of thiadiazole ring was confirmed.

**Scheme 3:**
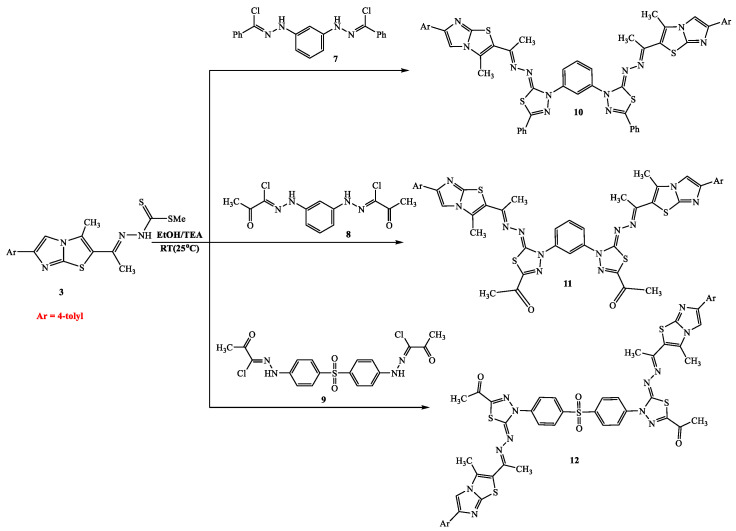
Synthesis of *bis*-thiadiazole derivatives **10**–**12**.

*Bis*-heterocycles have been attracting much attention because of their diverse biological and pharmaceutical activities, such as DNA-binding applications, tuberculostatic, antibacterial, non-Competitive mGluR1 antagonists, antifungal, antidepressant, antitumor activity and antiviral activity [18,19,20,21,33,39]. In view of these reports, and in continuation with our research line in the synthesis of bioactive *bis*-heterocyclic compounds [17,22,23,24,25,26], we are exploring the synthesis of functionalized *bis*-thiadiazoles. Thus, the reaction of compound **3** with *bis*-hydrazonoylchlorides **7**–**9** in refluxing DMF in the presence of trimethylamine proceeded smoothly to give *bis*-thiadiazoles **10**–**12**, respectively (Scheme 3). The products were elucidated by means of their elemental analysis and spectral data. For example, the ^1^H-NMR spectrum of compound **10** showed four singlet signals at δ = 2.38, 2.42, 2.58, and 7.97 ppm, which were assigned for 3 methyl groups and the imidazole proton, in addition to 22 aromatic protons at δ = 7.30–7.62 ppm.

### 2.2. Anti-Proliferative Activity

The results of anti-proliferative activity were calculated based on the IC_50_ (inhibitory concentration 50%), the concentration of a tested compound which inhibits 50% of the cells population. IC_50_ esteems were determined for each examination independently and mean qualities ± SD are introduced in Table 1. The movement of every subordinate at every fixation was tried in three-fold in a solitary test; which was rehashed 3–5 times. The aftereffects of the investigations on against proliferative action of tried mixes are summed up in Table 1

The results stated in Table 1 showed that many compounds exhibited good anti-proliferative activity against hepatic cancer cell lines (HepG2) with no toxicity on normal cell lines. It showed that compound **12** was the most active against hepatic cancer and normal cell lines (IC_50_ value of 12.73 ± 1.36 and 8.34 ± 1.81 μg/mL**,** respectively), while IC_50_ of Doxorubicin were = 3.56 ± 0.46 and 3.21 ± 0.32 μg/mL, respectively.

### 2.3. Computational Studies

**Figure 1:**
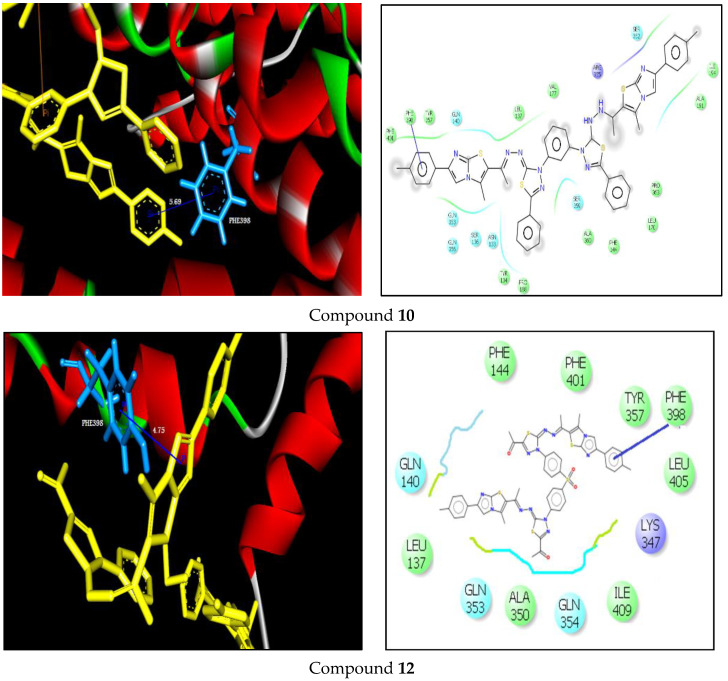
Intermolecular interactions between the best docked molecules (**10** and **12**) and GPC-3 protein. (Left) 3-Dimensional representation of GPC3-ligand complexes. The binding residues are shown in cyan stick and the hydrogen bonds in Å are represented in green dotted lines. π-interactions are shown in blue lines. The ligand molecules are shown in yellow stick model. (Right) 2-Dimensional representation; the amino acids are shown in 3 letter code, and H-bonds in green dotted lines. π-interactions are shown in blue lines.

In the present study, the thiadiazole compounds were evaluated through in silico molecular docking studies using the PyRx tool. By using a protein BLAST search, 4ACR was chosen as a best template for generating the 3D model of GPC-3 protein. Twenty models were generated and the model with lowest modeler objective function and least restraint violation was selected for further studies. The homologue model of the target consists of 27 helices and 1 strand as obtained from PDBsum server [27]. The binding site prediction tools declared that the residues SER136, PHE144, ARG297, GLN356, TYR357, PHE398, PHE401 and TYR408 were crucial for GPC-3 protein to bind with the compounds. The grid with dimensions 60 Å × 60 Å × 60 Å was generated around active site region. The compounds were computationally docked with the active site of the target protein to explore the inhibitory actions. The docking studies against the target protein revealed that all the compounds were suitably docked on the protein with good binding affinity −10.30 to −6.90 kcal·mol^−1^. A list of promising compounds displaying high binding affinities towards GPC-3 is reported in Table 2. Among the synthesized compounds, derivative **12** exhibited the best binding affinity of value −10.30 kcal·mol^−1^. Different bonding interactions such as hydrogen bonding and pi-stacking are tabulated in Table 2. The minimum binding affinity indicates stronger binding and comfortable docking conformation [28,29,30]. The interactions of the docked compounds-target complexes are visualized using Accelrys discovery studio software [31]. Figure 1 represents the 3D structures of the best docked compounds (**10** and **12**)-protein complexes. The other docked compounds are included in Supplementary Materials as Figure S1. The binding pockets of GPC-3 protein are shown in a cyan-colored stick model and the ligands in a yellow one. The hydrogen bonds are represented in green dotted lines, and pi- interactions are shown in blue lines. The Schrodinger Maestro software [32] was used to generate the images of the two dimensional (2D) structures of docking between compounds and the receptor. Compound **3** interacts with the protein at ARG197, forming two pi-cation interactions at distance of 6.42 and 6.49 Å. This interaction is due to arginine contains positively charged guanidinium group H_2_N-C(=NH)-NH– that is involved in forming pi-cation interaction with thiazole moiety in compound **3**. Compounds **6a**–**6c** exhibited an H-bond (O-H⋯O) with SER136 at 1.97, 1.99 and 2.53 Å respectively. Due to the presence of methyl group at position -4 on phenyl ring, compound **6a** exhibited the highest binding energy as compared with the other derivatives, whereas the methoxy and chlorine groups at the same position in compounds **6b** and **6c** exhibited least binding energy of −9.11 and −8.17 kcal·mol^−1^ respectively. Introducing electron withdrawing groups as chlorine atoms on phenyl ring causes the moderate activity and decreases of the IC_50_ as is shown for compound **6c** (Table 1). On the other hand, the electron donating groups on the benzene ring like methoxy enhances the activity, as shown in compound **6b** [40]. In addition, compounds **6d**–**6f** formed an H-bond with GLN356 (N-H⋯S), at distances of 2.10, 2.42 and 2.71 Å respectively. Compounds **6g** and **6i** formed pi-pi stacking with TYR357, PHE144 and PHE401, at distances of 4.45, 4.81, 4.82 and 4.02 Å respectively, while compound **6h** exhibited H-bond with SER136 (O-H⋯O) at distances of 2.90 Å. The effect of electron withdrawing and/or donating groups is shown in Table 2. Finally, compounds **10**, **11** and **12** showed pi-pi interactions with the target protein through PHE398 and TYR408. On the other hand, Doxorubicin which is used as a reference has the binding affinity (−8.6 kcal·mol^−1^) closer to the synthesized compounds and declared 4 H-bond interactions with TYR255, CYS401 and PRO267, respectively. The results showed that phenylalanine (PHE) and tyrosine (TYR) residues contain an aromatic ring that is involved in the formation of the pi-pi interaction with thiadiazole moiety of compound **6g**, **6i**, **10**, **11** and **12**. Unmistakably, imidazo[2,1-*b*]thiazole and thiadiazole analogs are seen to be normal pharmacophore bunches which cooperate with the coupling deposits of GPC-3 protein through different connections as spoken to in Table 2. It is likewise seen that, all solid associations of the mixes with GPC-3 protein had happened through hydrogen and additionally non-covalent bonds, for example, pi-stacking. Lower restricting energy and more non-covalent bonds inferred the best restricting dependability and inhibitory quality of the incorporated mixes against hepatocellular carcinoma. Medication similarity scores of the screened thiadiazole mixes were separated by the in silico forecast of ADME, utilizing admet SAR. The goal of in silico ADME was to investigate the disposition behavior of the compounds in human body. From the results in Table 3, it is clear that all the newly synthesized compounds can be absorbed by the human intestines, non-carcinogenic, safe regarding toxicity and have good drug score values. In addition, such compounds were found to obey Lipinski’s rule of five [41]. Topological polar surface area, number of hydrogen bonds donor and acceptor, and number of rotatable bonds are within an acceptable range. The ligand compounds with the acceptable ADMET properties are considered to be as prominent anticancer drug candidates [42]. The physicochemical properties of the title compounds (**3**–**12**) are shown in Table 4.

The molecules (**3**–**12**) with the best binding affinity are represented with docking interactions H-bonding, pi–pi, and pi-cation.

The pharmacokinetic properties of the molecules (**3**–**12**) which form docked complexes with GPC-3 protein are evaluated by admetSAR.

## 3. Conclusions

In this investigation, compound **3** was used as a key precursor for the synthesis of new thiadiazole derivatives incorporating imidazo[2,1-*b*]thiazole in good yields. The synthetic thiadiazole compounds had shown good binding affinities compared to the standard drug and interacted significantly with the binding site region of the target GPC-3, through non-covalent interactions. All the newly synthesized compounds were tested for their anti-proliferative activity against hepatic cancer cell lines (HepG2); compound **12** was the most active against hepatic cancer and normal cell lines (IC_50_ value of 12.73 ± 1.36 and 8.34 ± 1.81 μg/mL, respectively), while IC_50_ of doxorubicin were = 3.56 ± 0.46 and 3.21 ± 0.32 μg/mL, respectively. Hence, the output of the presented research holds a promising insight in synthesis of novel compounds with significant biological activities as anti-hepatic cancer agents. Furthermore, based on the computational studies’ results, the newly synthetic derivatives could be regarded as efficient drug candidates for further molecular development of HCC agents.

## 4. Experimental

### 4.1. Experimental Instrumentation

All melting points were estimated on an electrothermal mechanical assembly and were uncorrected. IR spectra were recorded (KBr circles) on a Shimadzu FT-IR 8201 PC spectrophotometer (Kyoto, Japan). 1H-NMR spectra were recorded in (CD3)2SO arrangements on BRUKER 400 FT-NMR framework spectrometer (Karlsruhe, Germany) and substance shifts were communicated in ppm units utilizing TMS as an inward reference. Mass spectra were recorded on a GC-MS QP1000 EX Shimadzu (Kyoto, Japan). Essential examinations were done at the Microanalytical Center of Cairo University.

Imidazo[2,1-*b*]thiazoles **1** [43,44] methyl hydrazine carbodithioate **2** [45] and hydrazonoyl halides **4a**–**j** and **7**–**9** [46,47,48] were prepared as previously reported.

### 4.2. Synthesis of methyl 2-(1-(3-methyl-6-(p-tolyl)imidazo[2,1-b]thiazol-2-yl)ethylidene) hydrazinecarbodithioate *(**3**)*

To a solution of 1-(3-methyl-6-(*p*-tolyl)imidazo[2,1-*b*]thiazol-2-yl)ethan-1-one **1** (l0 mmol) in 2-propanol (20 mL), methyl hydrazinecarbodithioate **2** (1.22 g, 10 mmol) was included. The blend was mixed at room temperature for 2 h. The strong item was sifted through, recrystallized from ethanol to afford 3. Yellowish white solid (2.5 g, 67%), m.p. 182–184 °C, IR (KBr): ν 3419 (NH), 3053, 2924 (C-H), 1627 (C=N) cm^−1^; ^1^H-NMR (DMSO-*d*_6_): δ 2.36 (s, 3H, CH_3_), 2.43 (s, 3H, CH_3_), 2.48 (s, 3H, CH_3_), 2.51 (s, 3H, SCH_3_), 7.21–7.60 (m, 4H, Ar-H), 9.09 (m, 1H, imidazole proton), 12.60 (br s, 1H, NH); ^13^C-NMR (CDCl_3_): δ 12.9, 14.5, 18.0, 21.2, 116.3, 128.3, 128.5, 129.2, 129.4, 133.0, 133.7, 135.7, 139.7, 143.0, 144.6, 151.5, 199.5; MS *m*/*z* (%): 374 (M^+^, 14), 344 (12), 260 (11), 213 (12), 158 (42), 126 (65), 94 (68), 76 (100). Anal. Calcd. for C_17_H_18_N_4_S_3_ (374.55) (%): C, 54.51; H, 4.84; N, 14.96. Found (%): C, 54.27; H, 4.80; N, 14.77.

### 4.3. Synthesis of 2-((1-((1,3,4-thiadiazolylidene)hydrazono)ethyl)-3-methyl-6-phenylimidazo[2,1-b]thiazoles *(**6a**–**i**)*

General procedure: To a blend of methyl carbodithioate **3** (0.37 g, 1 mmol) and the proper hydrazonoyl halide **4a**–**i** (1 mmol) in ethanol (20 mL), triethylamine (0.5 mL) was included, the blend was then mixed at room temperature for 2–4 h (checked with TLC). The subsequent strong was gathered and recrystallized from the best possible dissolvable to give the comparing 1,3,4-thiadiazolines **6a**–**i**. The items with their physical constants are recorded underneath.

*1-(5-((1-(3-Methyl-6-(p-tolyl)imidazo[2,1-b]thiazol-2-yl)ethylidene)hydrazono)-4-(p-tolyl)-4,5-dihydro-1,3,4-thiadiazol-2-yl)ethanone* (**6a**). Red solid (0.37 g, 75%), m.p. 220–222 °C (EtOH); IR (KBr): ν 3082, 2951 (C-H), 1653 (C=O), 1585 (C=N) cm^−1^; ^1^H-NMR (DMSO-*d*_6_): δ 2.29 (s, 3H, CH_3_), 2.34 (s, 3H, CH_3_), 2.35 (s, 3H, CH_3_), 2.36 (s, 3H, CH_3_), 2.44 (s, 3H, CH_3_), 7.20–7.90 (m, 9H, Ar-H and imidazole proton); ^13^C-NMR (CDCl_3_): δ 13.0, 14.5, 21.3, 21.5, 25.1, 116.4, 122.6, 122.8, 128.1, 128.3, 129.0, 129.2, 129.6, 129.8, 132.5, 133.0, 133.8, 135.8, 139.4, 139.8, 143.0, 144.6, 150.6, 151.5, 160.4, 190.4; MS *m*/*z* (%): 500 (M^+^, 8), 382 (16), 261 (100), 208 (30), 132 (35), 106 (60), 91 (50). Anal. Calcd. for C_26_H_24_N_6_OS_2_ (500.64) (%): C, 62.38; H, 4.83; N, 16.79. Found (%): C, 62.27; H, 4.92; N, 16.55.

*1-(4-(4-Methoxyphenyl)-5-((1-(3-methyl-6-(p-tolyl)imidazo[2,1-b]thiazol-2-yl)ethylidene) hydrazono)-4,5-dihydro-1,3,4-thiadiazol-2-yl)ethanone* (**6b**). Red solid (0.36 g, 70%), m.p. 216–218 °C (EtOH); IR (KBr): ν 3054, 2929 (C-H), 1671(C=O), 1579 (C=N) cm^−1^; ^1^H-NMR (DMSO-*d*_6_): δ 2.32 (s, 3H, CH_3_), 2.34 (s, 3H, CH_3_), 2.49 (s, 3H, CH_3_), 2.54 (s, 3H, CH_3_), 3.82 (s, 3H, OCH_3_), 7.08–7.11 (m, 4H, Ar-H), 7.28 (s, 1H, imidazole proton), 7.86–7.89 (m, 4H, Ar-H); ^13^C-NMR (CDCl_3_): δ 12.9, 14.5, 21.2, 25.0, 55.4, 114.2, 114.4, 116.3, 128.3, 128.5, 128.7, 128.9, 129.2, 129.4, 133.0, 133.7, 135.7, 139.3, 139.7, 143.0, 144.6, 150.5, 151.5, 156.8, 160.4, 190.3; MS *m*/*z* (%): 516 (M^+^, 6), 466 (11), 402 (100), 386 (17), 359 (49), 230 (28), 166 (30), 140 (58), 72 (47), 66 (73). Anal. Calcd. for C_26_H_24_N_6_O_2_S_2_ (516.64) (%): C, 60.44; H, 4.68; N, 16.27. Found (%): C, 50.52; H, 5.08; N, 27.57.

*1-(4-(4-Chlorophenyl)-5-((1-(3-methyl-6-(p-tolyl)imidazo[2,1-b]thiazol-2-yl)ethylidene) hydrazono)-4,5-dihydro-1,3,4-thiadiazol-2-yl)ethanone* (**6c**). Orange solid (0.39 g, 75%), m.p. 192–194 °C (EtOH); IR (KBr): ν 3087, 2967 (C-H), 1653 (C=O), 1583 (C=N) cm^−1^; ^1^H-NMR (DMSO-*d*_6_): δ 2.36 (s, 3H, CH_3_), 2.37 (s, 3H, CH_3_), 2.42 (s, 3H, CH_3_), 2.58 (s, 3H, CH_3_), 7.33 (s, 1H, imidazole proton), 7.61–7.64 (m, 4H, Ar-H), 8.08–8.11 (m, 4H, Ar-H); ^13^C-NMR (CDCl_3_): δ 12.9, 14.5, 21.2, 25.0, 116.3, 124.2, 124.4, 128.3, 128.5, 129.0, 129.2, 129.4, 129.7, 129.9, 133.0, 133.7, 135.7, 139.3, 139.7, 143.0, 144.6, 150.5, 151.5, 160.3, 190.3; MS *m*/*z* (%): 523 (M^+^ + 2, 3), 521 (M^+^, 10), 451 (10), 422 (14), 321(13), 281 (100), 228 (32), 152 (37), 126 (55), 99 (47), 75 (63). Anal. Calcd. for C_25_H_21_ClN_6_OS_2_ (521.06) (%): C, 57.63; H, 4.06; N, 16.13. Found (%): C, 57.75; H, 4.01; N, 16.04.

*Ethyl 5-((1-(3-methyl-6-(p-tolyl)imidazo[2,1-b]thiazol-2-yl)ethylidene)hydrazono)-4-phenyl-4,5-dihydro-1,3,4-thiadiazole-2-carboxylate* (**6d**). Yellow solid (0.35 g, 69%), m.p. 235–237 °C (EtOH); IR (KBr): ν 3088, 2927 (C-H), 1733 (C=O), 1579 (C=N) cm^−1^; ^1^H-NMR (DMSO-*d*_6_): δ 1.33 (t, 3H, *J* = 7.2 Hz, CH_3_CH_2_), 2.35 (s, 3H, CH_3_), 2.36 (s, 3H, CH_3_), 2.51 (s, 3H, CH_3_), 4.39 (q, 2H, *J* = 7.2 Hz, CH_3_CH_2_), 7.30 (s, 1H, imidazole proton), 7.37–7.98 (m, 9H, Ar-H); ^13^C-NMR (CDCl_3_): δ 13.0, 14.1, 14.5, 21.3, 62.5, 116.4, 121.6, 121.8, 124.7, 128.1, 128.3, 129.0, 129.2, 129.6, 129.8, 133.0, 133.8, 135.8, 139.4, 139.8, 143.0, 144.6, 151.5, 159.7, 160.4, 163.9; MS *m*/*z* (%): 516 (M^+^, 18), 485 (9), 431 (22), 401 (34), 333 (31), 259 (82), 242 (75), 178 (41), 170 (100), 144 (69), 103 (92), 65 (58). Anal. Calcd. for C_26_H_24_N_6_O_2_S_2_ (516.64) (%): C, 60.44; H, 4.68; N, 16.27. Found (%): C, 60.30; H, 4.54; N, 16.08.

*Ethyl 5-((1-(3-methyl-6-(p-tolyl)imidazo[2,1-b]thiazol-2-yl)ethylidene)hydrazono)-4-(p-tolyl)-4,5-dihydro-1,3,4-thiadiazole-2-carboxylate* (**6e**). Brown solid (0.37 g, 70%), m.p. 117–119 °C (EtOH); IR (KBr): ν 3081, 2919 (C-H), 1713 (C=O), 1587 (C=N) cm^−1^; ^1^H-NMR (DMSO-*d*_6_): δ 1.32 (t, 3H, *J* = 7.2 Hz, CH_3_CH_2_), 2.23 (s, 3H, CH_3_), 2.34 (s, 3H, CH_3_), 2.40 (s, 3H, CH_3_), 2.48 (s, 3H, CH_3_), 4.38 (q, 2H, *J* = 7.2 Hz, CH_3_CH_2_), 7.08–7.83 (m, 9H, Ar-H and imidazole proton); ^13^C-NMR (CDCl_3_): δ 12.9, 14.1, 14.5, 21.2, 21.4, 62.5, 116.3, 122.8, 123.0, 128.3, 128.5, 129.2, 129.4, 129.8, 130.0, 132.4, 139.3, 139.7, 133.7, 133.0, 135.7, 143.0, 144.6, 151.5, 159.7, 160.3, 163.8; MS *m*/*z* (%): 530 (M^+^, 3), 468 (8), 416 (9), 364 (61), 318 (17), 272 (23), 258 (58), 218 (33), 174 (27), 149 (16), 107 (92),77 (100). Anal. Calcd. for C_27_H_26_N_6_O_2_S_2_ (530.66) (%): C, 61.11; H, 4.94; N, 15.84. Found (%): C, 61.04; H, 4.77; N, 15.79.

*Ethyl 4-(4-bromophenyl)-5-((1-(3-methyl-6-(p-tolyl)imidazo[2,1-b]thiazol-2-yl)ethylidene) hydrazono)-4,5-dihydro-1,3,4-thiadiazole-2-carboxylate* (**6f**). Yellow solid (0.39 g, 66%), m.p. 156–158 °C (EtOH); IR (KBr): ν 3087, 2966 (C-H), 1703 (C=O), 1589 (C=N) cm^−1^; ^1^H-NMR (DMSO-*d*_6_): δ 1.33 (t, 3H, *J* = 7.2 Hz, CH_3_CH_2_), 2.35 (s, 3H, CH_3_), 2.37 (s, 3H, CH_3_), 2.49 (s, 3H, CH_3_), 4.39 (q, 2H, *J* = 7.2 Hz, CH_3_CH_2_), 7.33 (s, 1H, imidazole proton), 7.72–7.75 (m, 4H, Ar-H), 7.95–7.98 (m, 4H, Ar-H); ^13^C-NMR (CDCl_3_): δ 12.9, 14.1, 14.5, 21.2, 62.5, 116.3, 117.9, 121.6, 121.8, 128.3, 128.5, 129.2, 129.4, 131.8, 132.0, 133.0, 133.7, 135.7, 139.3, 139.7, 143.0, 144.6, 151.5, 159.7, 160.3, 163.8; MS *m*/*z* (%): 597 (M^+^+2, 4), 595 (M^+^, 6), 422 (24), 382 (38), 339 (31), 281 (19), 261 (22), 208 (22), 152 (14), 132 (20), 77 (100). Anal. Calcd. for C_26_H_23_BrN_6_O_2_S_2_ (595.53) (%): C, 52.44; H, 3.89; N, 14.11. Found (%): C, 52.31; H, 3.82; N, 14.04.

*5-((1-(3-Methyl-6-(p-tolyl)imidazo[2,1-b]thiazol-2-yl)ethylidene)hydrazono)-N,4-diphenyl-4,5-dihydro-1,3,4-thiadiazole-2-carboxamide* (**6g**). Yellow solid (0.38 g, 68%), m.p. 209–211 °C (EtOH); IR (KBr): ν 3433 (NH), 3050, 2931 (C-H), 1662 (C=O), 1594 (C=N) cm^−1^; ^1^H-NMR (DMSO-*d*_6_): δ 2.37 (s, 3H, CH_3_), 2.40 (s, 3H, CH_3_), 2.51 (s, 3H, CH_3_), 7.30–8.23 (m, 15H, Ar-H and imidazole proton), 10.61 (br s, 1H, NH); ^13^C-NMR (CDCl_3_): δ 12.9, 14.5, 21.2, 116.3, 120.4, 120.6, 121.8, 122.0, 124.7, 124.9, 128.3, 128.5, 129.0, 129.1, 129.3, 129.4, 129.5, 129.6, 133.0, 133.7, 135.7, 137.4, 139.3, 139.7, 143.0, 144.6, 151.5, 160.3, 163.2, 163.8; MS *m*/*z* (%): 563 (M^+^, 37), 510 (17), 461 (43), 425 (52), 378 (30), 326 (25), 300 (99), 274 (95), 233 (47), 192 (64), 127 (29), 91 (25), 76 (100). Anal. Calcd. for C_30_H_25_N_7_OS_2_ (563.70) (%): C, 63.92; H, 4.47; N, 17.39. Found (%): C, 63.83; H, 4.40; N, 17.27.

*5-((1-(3-Methyl-6-(p-tolyl)imidazo[2,1-b]thiazol-2-yl)ethylidene)hydrazono)-4-(4-nitro phenyl)-N-phenyl-4,5-dihydro-1,3,4-thiadiazole-2-carboxamide* (**6h**). Red solid (0.42 g, 70%), m.p. 241–243 °C (EtOH); IR (KBr): ν 3433 (NH), 3051, 2920 (C-H), 1660 (C=O), 1626 (C=N) cm^−1^; ^1^H-NMR (DMSO-*d*_6_): δ 2.38 (s, 3H, CH_3_), 2.46 (s, 3H, CH_3_), 2.50 (s, 3H, CH_3_), 7.19–7.77 (m, 10H, Ar-H and imidazole proton), 8.40 (d, *J* = 9.3 Hz, 2H, Ar-H), 8.64 (d, *J* = 9.3 Hz, 2H, Ar-H), 10.72 (br s, 1H, NH); ^13^C-NMR (CDCl_3_): δ 13.0, 14.5, 21.3, 116.4, 117.3, 117.5, 120.2, 120.4, 123.2, 123.4, 124.7, 128.1, 128.3, 129.0, 129.2, 129.3, 129.5, 133.0, 133.8, 135.8, 137.5, 139.4, 139.8, 140.5, 143.0, 144.6, 151.5, 160.4, 163.2, 163.9; MS *m*/*z* (%): 608 (M^+^, 13), 575 (35), 516 (24), 432 (17), 368 (46), 264 (34), 180 (25), 156 (92), 122 (21), 93 (100). Anal. Calcd. for C_30_H_24_N_8_O_3_S_2_ (608.69) (%): C, 59.20; H, 3.97; N, 18.41. Found (%): C, 59.12; H, 3.84; N, 18.29.

*2-(1-((3,5-Diphenyl-1,3,4-thiadiazol-2(3H)-ylidene)hydrazono)ethyl)-3-methyl-6-(p-tolyl) imidazo[2,1-b]thiazole* (**6i**). Gray solid (0.35 g, 68%), m.p. 273–275 °C (EtOH); IR (KBr): ν 3051, 2920 (C-H), 1626 (C=N) cm^−1^; ^1^H-NMR (DMSO-*d*_6_): δ 2.35 (s, 3H, CH_3_), 2.40 (s, 3H, CH_3_), 2.49 (s, 3H, CH_3_), 7.42–7.70 (m, 15H, Ar-H and imidazole proton); ^13^C-NMR (CDCl_3_): δ 13.0, 14.5, 21.3, 116.4, 121.6, 121.8, 124.7, 125.7, 125.9, 128.1, 128.3, 128.9, 129.0, 129.0, 129.2, 129.2, 129.4, 129.4, 130.4, 133.0, 133.8, 135.8, 139.4, 139.8, 143.0, 144.6, 151.5, 160.4, 175.7; MS *m*/*z* (%): 520 (M^+^, 10), 479 (16), 445 (18), 369 (20), 294 (17), 226 (100), 217 (10), 183 (67), 152 (22), 108 (15), 77 (36). Anal. Calcd. for C_29_H_24_N_6_S_2_ (520.67) (%): C, 66.90; H, 4.65; N, 16.14. Found (%): C, 66.82; H, 4.47; N, 16.05.

### 4.4. Synthesis of bis-thiadiazoles **10**, **11** and **12**

To a blend of methyl carbodithioate **3** (0.74 g, 2 mmol) and the proper *bis*-hydrazonoyl halide **7**–**9** (1 mmol) in ethanol (20 mL), triethylamine (0.5 mL) was included, the blend was then mixed at room temperature for 3 h. The subsequent strong was gathered and recrystallized from the correct dissolvable to give the comparing 1,3,4-thiadiazolines **10**–**12**. The items **10**–**12** along with their physical constants are recorded beneath.

*1,3-Bis(2-((1-(3-methyl-6-(p-tolyl)imidazo[2,1-b]thiazol-2-yl)ethylidene)hydrazono)-5-phenyl-1,3,4-thiadiazol-3(2H)-yl)benzene* (**10**). Orange solid (0.60 g, 63%), m.p. > 300 °C (Dioxane); IR (KBr): ν 3050, 2923 (C-H), 1583 (C=N) cm^−1^; ^1^H-NMR (DMSO-*d*_6_): δ 2.38 (s, 6H, 2CH_3_), 2.42 (s, 6H, 2CH_3_), 2.58 (s, 6H, 2CH_3_), 7.30–7.62 (m, 22H, Ar-H), 7.97 (s, 2H, imidazole proton); ^13^C-NMR (CDCl_3_): δ 12.7, 12.9, 14.3, 14.5, 21.2, 21.4, 103.4, 116.3, 116.5, 121.6, 121.8, 125.2, 125.4, 125.6, 125.9, 128.3, 128.5, 128.7, 128.8, 128.9, 129.0, 129.1, 129.2, 129.3, 129.4, 129.5, 129.6, 129.7, 129.8, 129.9, 130.4, 130.5, 133.0, 133.2, 133.5, 135.6, 135.7, 139.3, 139.5, 139.7, 133.7, 139.9, 143.0, 143.2, 144.4, 144.6, 151.5, 151.7, 160.1, 160.3, 175.7, 175.9; MS *m*/*z* (%): 963 (M^+^, 7), 818 (25), 653 (41), 478 (39), 322 (37), 244 (71), 125 (40), 77 (100). Anal. Calcd. for C_52_H_42_N_12_S_4_ (963.23) (%): C, 64.84; H, 4.39; N, 17.45. Found (%): C, 64.71; H, 4.34; N, 17.30.

*1,1’-(4,4’-(1,3-Phenylene)bis(5-((1-(3-methyl-6-(p-tolyl)imidazo[2,1-b]thiazol-2-yl) ethylidene)hydrazono)-4,5-dihydro-1,3,4-thiadiazole-4,2-diyl))diethanone* (**11**). Brown solid (0.53 g, 60%), m.p. 292–294 °C (Dioxane); IR (KBr): ν 3063, 2924 (C-H), 1679 (C=O), 1594(C=N) cm^−1^; ^1^H-NMR (DMSO-*d*_6_): δ 2.26 (s, 6H, 2CH_3_), 2.34 (s, 6H, 2CH_3_), 2.39 (s, 6H, 2CH_3_), 2.58 (s, 6H, 2CH_3_), 7.09–7.62 (m, 8H, Ar-H), 8.13 (m, 4H, Ar-H), 8.82 (s, 2H, imidazole proton); ^13^C-NMR (CDCl_3_): δ 12.7, 12.9, 14.3, 14.5, 21.2, 21.4, 25.0, 25.2, 103.4, 116.3, 116.5, 121.6, 121.8, 128.3, 128.5, 128.6, 128.7, 129.2, 129.3, 129.4, 129.6, 129.7, 133.0, 133.2, 133.7, 133.8, 135.7, 135.8, 139.3, 139.5, 139.6, 139.7, 143.0, 143.2, 144.6, 144.8, 150.5, 150.7, 151.5, 151.7, 160.3, 160.5, 190.3, 190.5; MS *m*/*z* (%): 895 (M^+^, 15), 711 (49), 637 (62), 428 (46), 316 (70), 232 (100), 157 (85), 103 (100), 63 (79). Anal. Calcd. for C_44_H_38_N_12_O_2_S_4_ (895.11) (%): C, 59.04; H, 4.28; N, 18.78. Found (%): C, 58.88; H, 4.14; N, 18.69.

*1,1’-(4,4’-(Sulfonylbis(4,1-phenylene))bis(5-((1-(3-methyl-6-(p-tolyl)imidazo[2,1-b]thiazol-2-yl)ethylidene)hydrazono)-4,5-dihydro-1,3,4-thiadiazole-4,2-diyl))diethanone* (**12**). Orange solid (0.64 g, 62%), m.p. > 300 °C (Dioxane); IR (KBr): ν 3033, 2926 (C-H), 1686 (C=O), 1585 (C=N) cm^−1^; ^1^H-NMR (DMSO-*d*_6_): δ 2.32 (s, 6H, 2CH_3_), 2.33 (s, 6H, 2CH_3_), 2.49 (s, 6H, 2CH_3_), 2.56 (s, 6H, 2CH_3_), 7.34–8.36 (m, 18H, Ar-H and imidazole proton); ^13^C-NMR (CDCl_3_): δ 12.9, 13.0, 14.5, 14.7, 21.2, 21.4, 25.0, 25.2, 116.3, 116.6, 118.0, 118.1, 118.3, 118.5, 127.4, 127.5, 127.6, 127.7, 128.1, 128.3, 128.5, 128.7, 129.2, 129.4, 129.6, 129.8, 132.3, 132.5, 133.0, 133.2, 133.6, 133.7, 135.3, 135.5, 139.3, 139.5, 139.7, 139.9, 143.0, 143.2, 144.6, 144.8, 150.5, 150.7, 151.5, 151.7, 160.3, 160.4, 190.3, 190.5; MS *m*/*z* (%):1035 (M+, 25), 850(10), 791 (9),711(12) 637 (62), 448 (26), 416 (71), 106 (100), 65 (77). Anal. Calcd. for C_50_H_42_N_12_O_4_S_5_ (1035.27) (%): C, 58.01; H, 4.09; N, 16.24. Found (%): C, 57.86; H, 4.01; N, 16.14.

### 4.5. Anti-Proliferative Activity Cells

As reported earlier by Feng M. et al. and Wang S. et al. [49,50], the in vitro analysis indicated that the HepG2 cell line had the highest protein expression level of GPC3 on the cell surface. Therefore, to determine new treatment strategies for hepatic cancer, we investigated the effect of GPC3 protein in the human hepatocellular carcinoma cell lines HepG2. In the present study, HepG2 cells have been selected as model for the effect of GPC3 protein expression on hepatocellular cancers cell growth.

Cell lines: Hep-G2 (human hepatic disease) and BALB/3T3 (murine fibroblast) are being kept up in the Institute of Cancer, Cairo, Egypt. All malignant growth cell lines were obtained from American Type Culture Collection (Rockville, Maryland, USA) and kept up in the Institute of Cancer. HepG2 cells were refined in Eagle’s minimum essential medium (EMEM) and supplemented with 10% FBS; DMEM and RPMI1640 were choices for that function also. Suction included new culture medium each 2–3 days. HepG2 cell multiplying time was 48 h. BALB/3T3 cell line was refined in DMEM and enhanced with 2 mM l-glutamine, 10% fetal ox-like serum (GE Healthcare, Logan, UT, USA). To entry cells, cell monolayer with 1× PBS were flushed twice and pre-warmed (37 °C) 0.05% Trypsin-EDTA arrangement was added to cover the lower part of the jar; brooded for 5–7 min. As cells withdrew, the trypsin was killed by including 4× volume of complete development medium with 10% FBS and delicately suspended the cells by pipetting. To abstain from clustering, the phones were not upset by shaking the jar while sitting tight for separation. Split cells 1:4 at regular intervals or 1:8 like clockwork. Societies had been brooded at 37 °C in a humidified climate with 5% CO_2_.

### 4.6. Compounds

All compounds were broken up in DMSO (stock arrangement 10 mg/mL) and consequently weakened in culture medium to arrive at the necessary fixations (going from 100 to 0.1 µg/mL).

### 4.7. An Anti-Proliferative Assay In Vitro

24 h before expansion of the tried exacerbates, the cells were plated in 96-well plates (Sarstedt, Germany) at thicknesses of 1 × 10^4^ cells per well. The examination was performed after 72 h introduction to shifting centralizations of the tried mixes. The in vitro cytotoxic impact of all mixes was analyzed utilizing the SRB measure.

### 4.8. Cytotoxic Test SRB

The subtleties of this strategy were depicted by Skehan et al. [44,45]. The cells were joined to the lower part of plastic wells by fixing them with cold half TCA (trichloroacetic corrosive, Sigma-Aldrich Chemie GmbH, Steinheim, Germany) on the head of the way of life medium in each well. The plates were hatched at 4 °C for 1 h and afterward washed multiple times with faucet water. The cell material fixed with TCA was recolored with 0.4% sulphorhodamine B (SRB, Sigma-Aldrich Chemie GmbH, Steinheim, Germany) disintegrated in 1% acidic corrosive for 30 min. Unbound color was taken out by flushing (multiple times) in 1% acidic corrosive. The protein-bound color was removed with 10 mMunbufferedTris base for assurance of the optical thickness (λ = 540 nm) in Synergy H4 multi-mode microplate peruser (BioTek Instruments, Winooski, VT, USA).

### 4.9. In Silico Studies

The computational based drug design approaches were used for prediction of drug-like molecules against hepatocellular carcinoma. Since the tertiary structure of human GPC-3 protein was not available in protein data bank (PDB), the structure was generated using the homology modeling technique. The amino acid sequence of human GPC-3 was retrieved from NCBI [51]. A basic local alignment search tool (BLASTP) [52] was used to identify the suitable template, which was used in generating the three dimensional (3D) model of the target. The homology modeling was carried out using protein structure modeling program: Modeller 9.11 [53]. The detection of ligand-binding site of the target protein plays an important role in drug design [54]. The active site region was predicted using computational prediction tools like MetaPocket2.0 [55] and ProBiS [56]. In silico docking studies of the synthetic compounds and human GPC3 protein were performed using PyRx bioinformatic docking tool [57]. In addition, doxorubicin was selected as a standard drug to compare the docking scores of synthesized compounds. The energy of the synthesized molecules was minimized in PyRx with default parameters (UFF force field), then docked flexibly to the target. The docking algorithms were applied to search for the best binding mode between the target and molecules. The criteria of filtration of ligands in each stage include good docking and good scoring poses. In silico ADME-Tox plays an important role in facilitating the selection of drug candidates [58]. The drug-likeness of the compounds was predicted using various software, such as admet SAR (http://lmmd.ecust.edu.cn/admetsar1/) and Mol inspiration (www.molinspiration.com) cheminformatics tools. The Lipinski rule of five gives information about the behavior of molecule in a living organism. The number of rotatable bonds is a good descriptor of the oral bioavailability of the drug. Topological polar surface area (TPSA) is a parameter for the prediction of drug transport properties.

## Figures and Tables

**Table 1:** Anti-proliferative activity of new derivatives towards hepatic cancer and normal cell lines.

Compound	HepG2 IC_50_ ± SD[µg/mL]	BALAB/3T3 IC_50_ ± SD[µg/mL]
**Doxorubicin**	3.56 ± 0.46	3.21 ± 0.32
**3**	Nd	Nd
**6a**	63.32 ± 12.83	25.09 ± 12.00
**6b**	59.20 ± 9.27	Nd
**6c**	Nd	Nd
**6d**	41.14 ± 8.32	Nd
**6e**	Nd	Nd
**6f**	57.52 ± 6.61	Nd
**6g**	33.24 ± 8.25	76.72 ± 4.14
**6h**	Nd	Nd
**6j**	19.93 ± 3.28	35.65 ± 1.27
**10**	23.51 ± 4.48	26.24 ± 3.21
**11**	Nd	Nd
**12**	12.73 ± 1.36	8.34 ± 1.81

Compounds were tested in concentration from 100 to 0.1 µg/mL; Nd: no detectable activity in the used concentrations; Concentration of DMSO: 1%.

**Table 2:** The binding affinity (kcal·mol^−1^) of various compounds **3**–**12** and reference drug with the target after molecular docking.

	Structure	Binding Affinity (kcal·mol^−1^)	Docked Complex (Amino Acid–Ligand) Interactions	Distance (Å)
Ref.	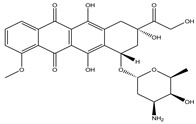	−8.60	H-bonds reference—TYR255 reference—TYR255 reference—CYS401 reference—PRO267	2.94 2.95 2.30 1.87
**3**	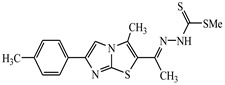	−6.90	π-cation interactions compound **3**—ARG197 compound **3**—ARG197	6.42 6.49
**6a**	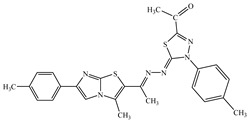	−9.40	H-bonds compound **6a**—-SER136	1.97
**6b**	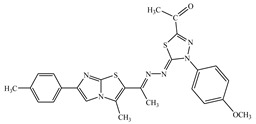	−9.11	H-bonds compound **6b**—SER136	1.99
**6c**	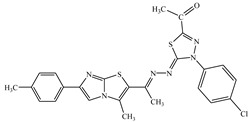	−8.17	H-bonds compound **6c**—SER136	2.53
**6d**	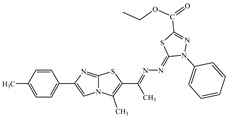	−8.15	H-bonds compound **6d**—GLN356	2.10
**6e**	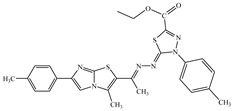	−8.11	H-bonds compound **6e**—GLN356	2.42
**6f**	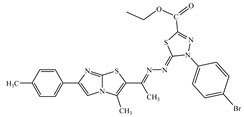	−7.91	H-bonds compound **6f**—GLN356	2.71
**6g**	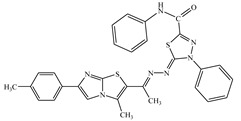	−9.30	π-π interactions compound **6g**—TYR357	4.45
**6h**	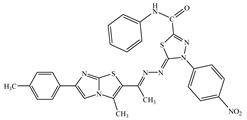	−8.52	H-bonds compound **6h**—SER136	2.90
**6i**	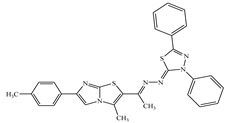	−9.03	π-π interactions compound **6i**—PHE144 compound **6i**—PHE401 compound **6i**—PHE401	4.81 4.82 4.02
**10**	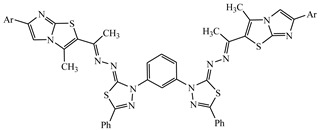	−9.52	π-π interactions compound **10**—PHE398	5.69
**11**	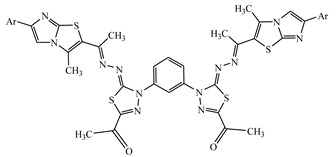	−9.01	π-π interactions compound **11**—TYR408	4.17
**12**	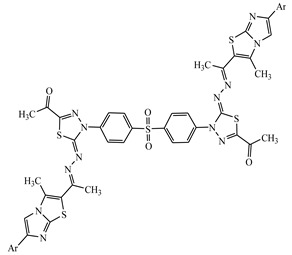	−10.30	π-π interactions compound **12**—PHE398	4.75

**Table 3:** List of ADMET properties of the newly synthesized molecules (**3**–**12**).

	Blood-Brain Barrier (BBB+)	Human Intestinal Absorption (HIA+)	Caco-2 Permeability (Caco2+)	AMES Toxicity	Carcino-Genicity
**3**	0.726	0.992	0.535	Nontoxic	Non carcinogenic
**6a**	0.898	1	0.559	Nontoxic	Non carcinogenic
**6b**	0.907	1	0.55	Nontoxic	Non carcinogenic
**6c**	0.872	1	0.541	Nontoxic	Non carcinogenic
**6d**	0.884	1	0.53	Nontoxic	Non carcinogenic
**6e**	0.884	1	0.53	Nontoxic	Non carcinogenic
**6f**	0.849	0.997	0.533	Nontoxic	Non carcinogenic
**6g**	0.857	0.996	0.556	Nontoxic	Non carcinogenic
**6h**	0.784	0.993	0.528	Nontoxic	Non carcinogenic
**6i**	0.9	0.994	0.588	Nontoxic	Non carcinogenic
**10**	0.9	0.994	0.588	Nontoxic	Non carcinogenic
**11**	0.9	0.997	0.536	Nontoxic	Non carcinogenic
**12**	0.532	0.95	0.567	Nontoxic	Non carcinogenic

**Table 4:** Physicochemical properties of the title compounds (**3**–**12**).

	TPSA (A^2^)	HBA	HBD	*n* Rotatable	Volume (A^3^)
**3**	41.70	4	1	5	319.26
**6a**	76.93	7	0	5	434.42
**6b**	86.17	8	0	6	443.40
**6c**	76.93	7	0	5	431.39
**6d**	86.17	8	0	7	443.64
**6e**	86.17	8	0	7	460.20
**6f**	86.17	8	0	7	461.53
**6g**	88.96	8	1	6	485.10
**6h**	134.78	10	1	7	508.44
**6i**	59.86	6	0	5	453.72
**10**	119.72	10	0	10	823.39
**11**	153.68	10	0	10	751.66
**12**	155.01	10	0	12	854.50

TPSA, topological polar surface area; HBA, number of hydrogen bond acceptors; HBD, number of hydrogen bond donors; *n* rotatable, number of rotatable bonds.

## Supplementary Materials

The following are available online, Figure S1: Intermolecular interactions between the other docked molecules and GPC-3 protein. (Left) 3-Dimensional representation. (Right) 2-Dimensional representation.
