# Supplementary material for: Synthesis, Molecular Docking Screening and Anti-Proliferative Potency Evaluation of Some New Imidazo[2,1-b]Thiazole Linked Thiadiazole Conjugates
Source: Molecules. 2020 Oct 28;25(21):4997. doi: 10.3390/molecules25214997 (PMC7663531; doi:10.3390/molecules25214997)
Supplement: Supplementary file 1 [file molecules-25-04997-s001.pdf]

Supplementary Materials

Synthesis, Molecular Docking Screening and Anti-Proliferative Potency Evaluation of Some New Imidazo[2,1-*b*]Thiazole Linked Thiadiazole Conjugates

Huda R.M. Rashdan[1*], Aboubakr H. Abdelmonsef [2], Ihsan A. Shehadi[3], Sobhi M. Gomha[4,5], Abdel Mohsen M. Soliman[6] and Huda K. Mahmoud[4]

1. *Chemistry of Natural and Microbial Products Department, Pharmaceutical and Drug Industries Research Division, National Research Centre, Giza, Egypt.
2. Chemistry Department, Faculty of Science, South Valley University, Qena 83523, Egypt.
3. Chemistry Department, Faculty of Science, University of Sharjah, Sharjah 27272, UAE.
4. Chemistry department, Faculty of Science, Cairo University, Giza 12613, Egypt.
5. Department of Chemistry, Faculty of Science, Islamic University in Almadinah Almonawara, Almadinah Almonawara, 42351, Saudi Arabia.
6. Therapeutic Chemistry Department, National Research Centre, Giza, Egypt.

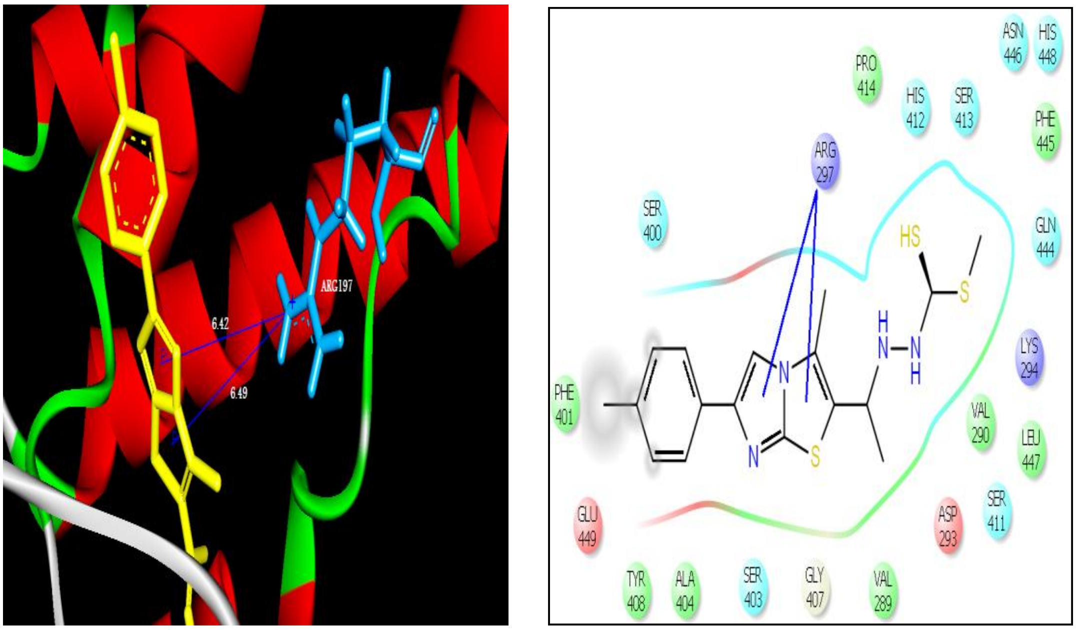

Compound **3**

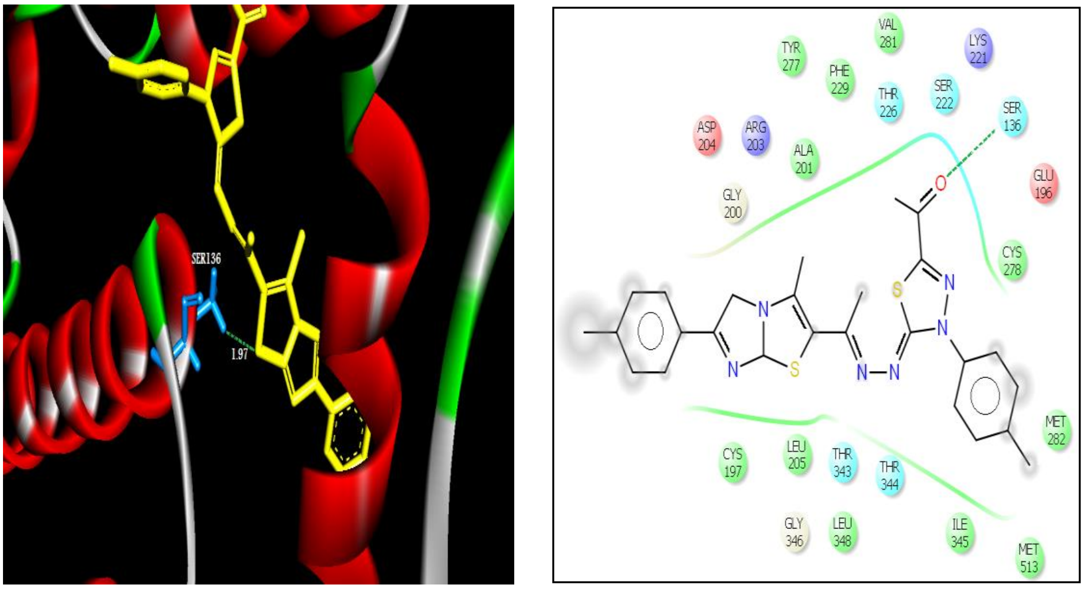

Compound **6a**

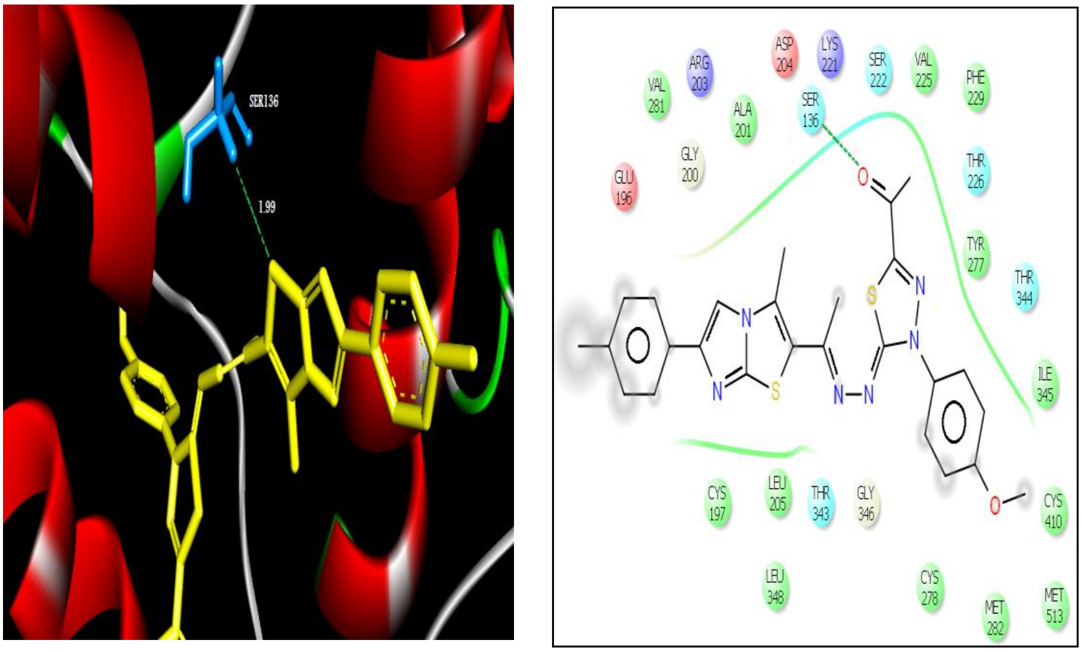

Compound **6b**

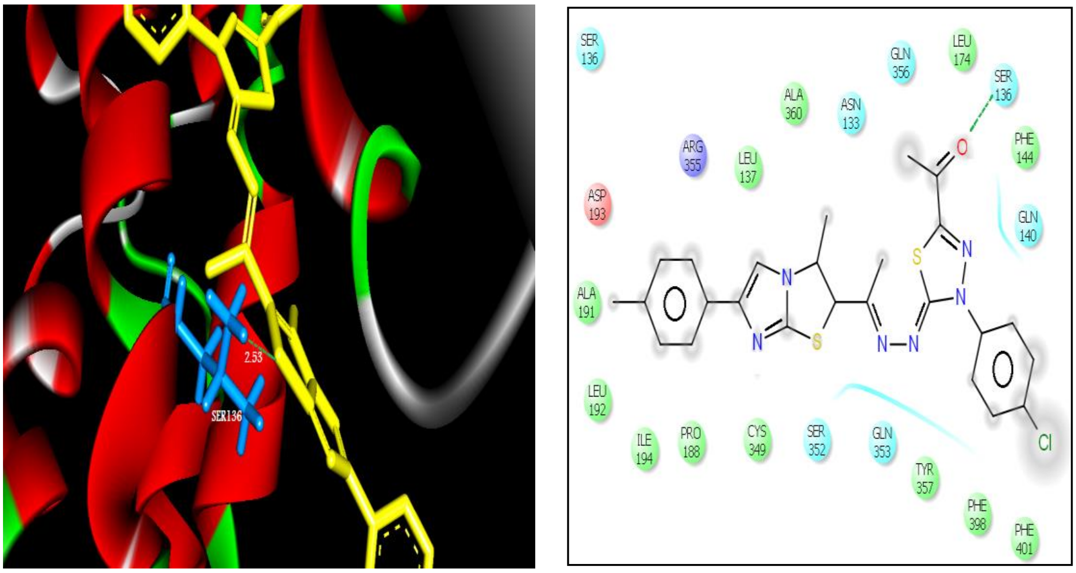

Compound **6c**

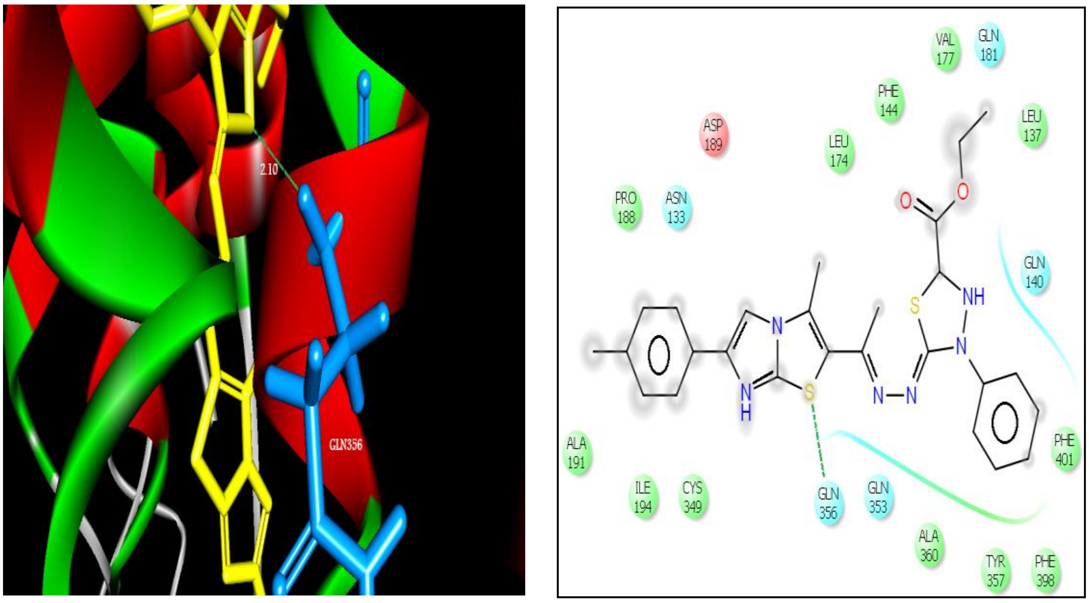

Compound **6d**

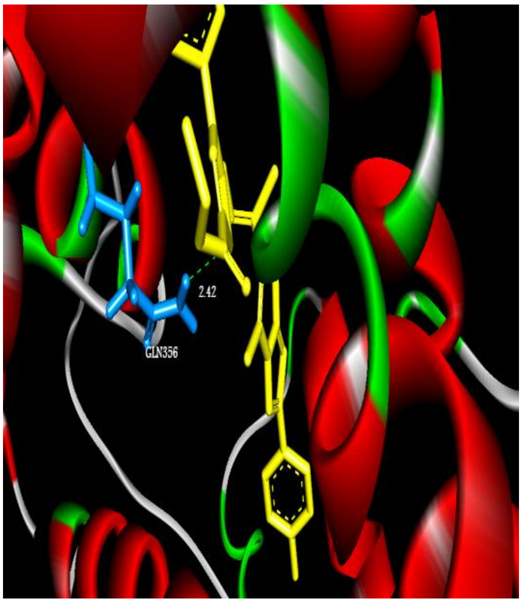

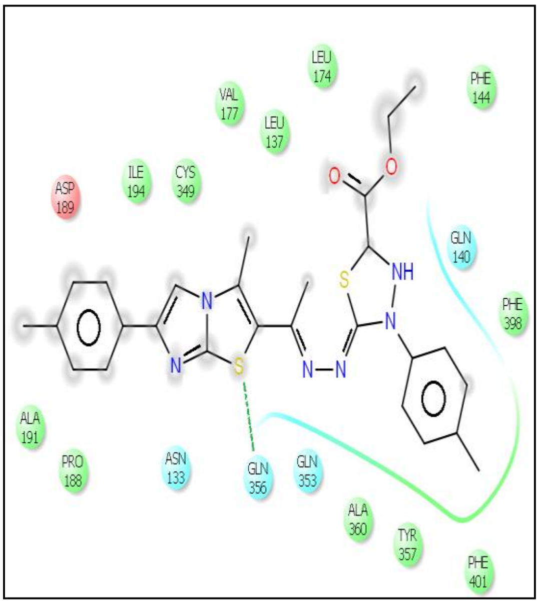

Compound **6e**

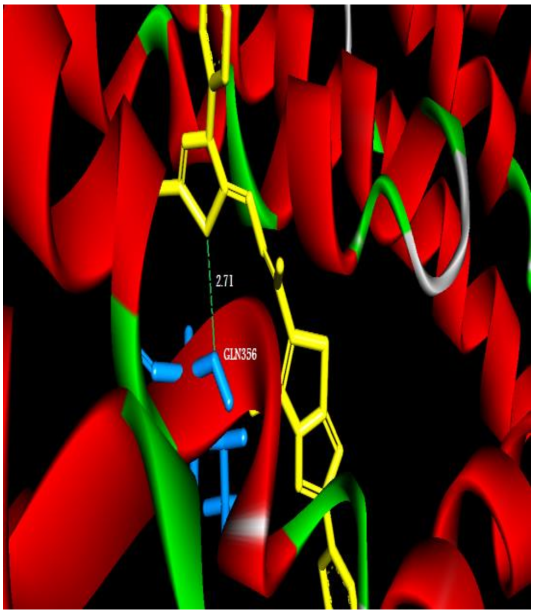

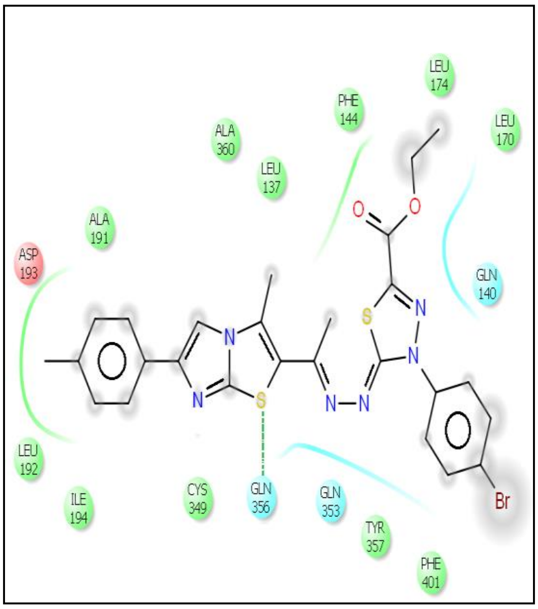

Compound **6f**

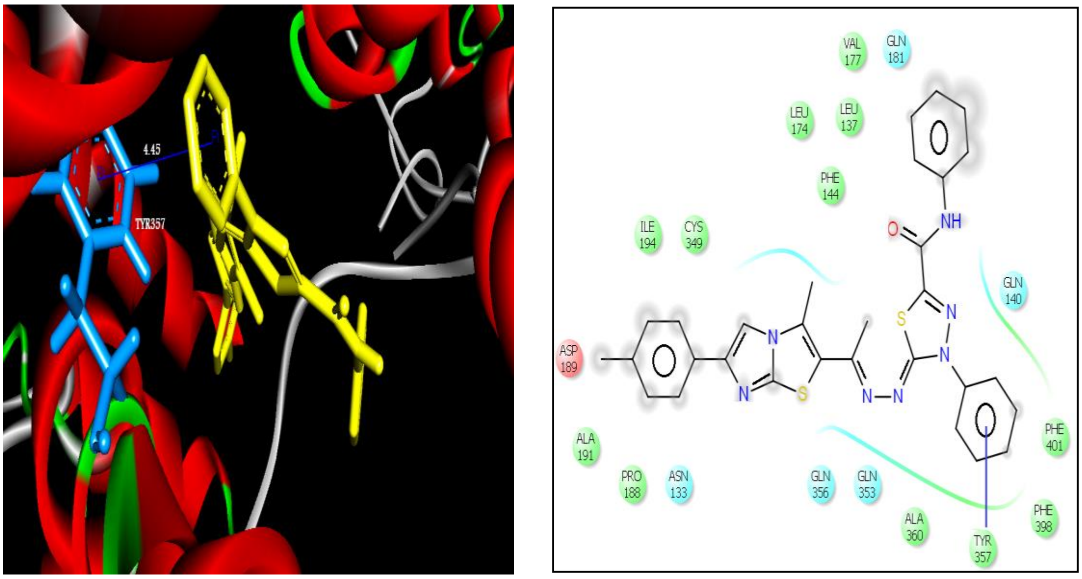

Compound **6g**

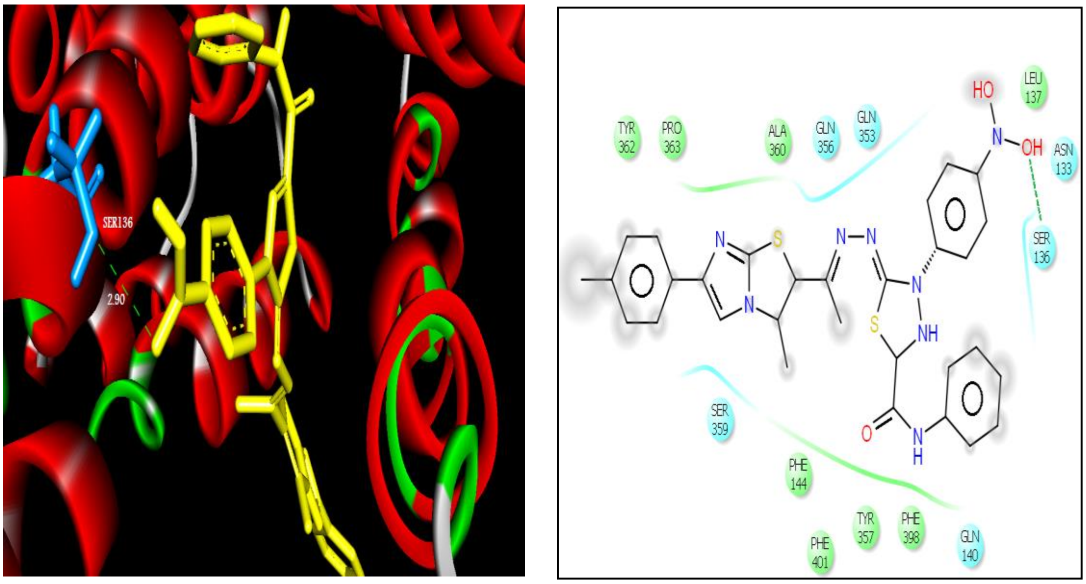

Compound **6h**

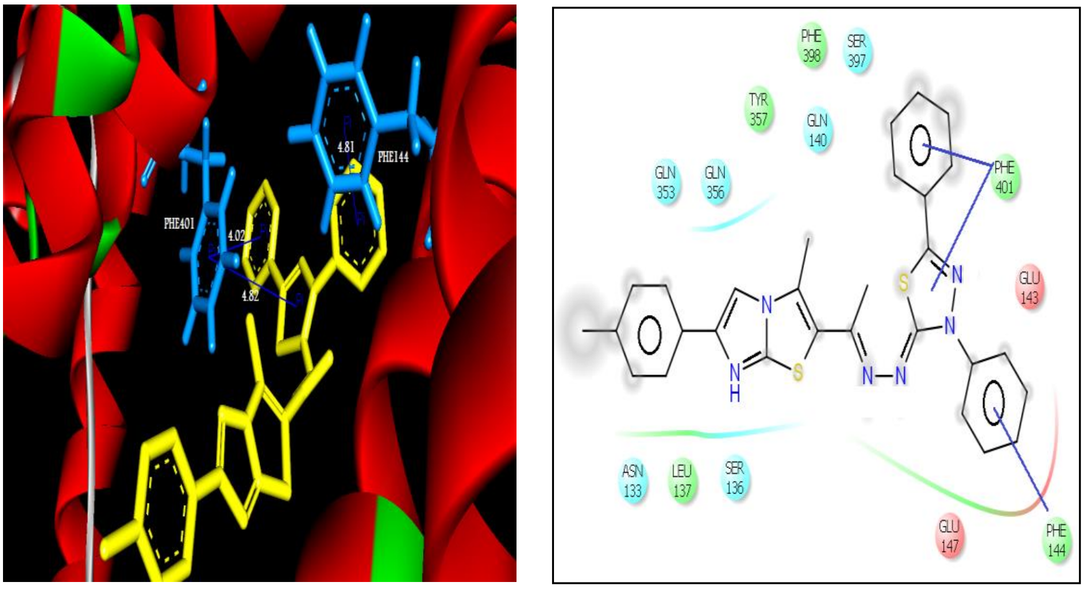

Compound **6i**

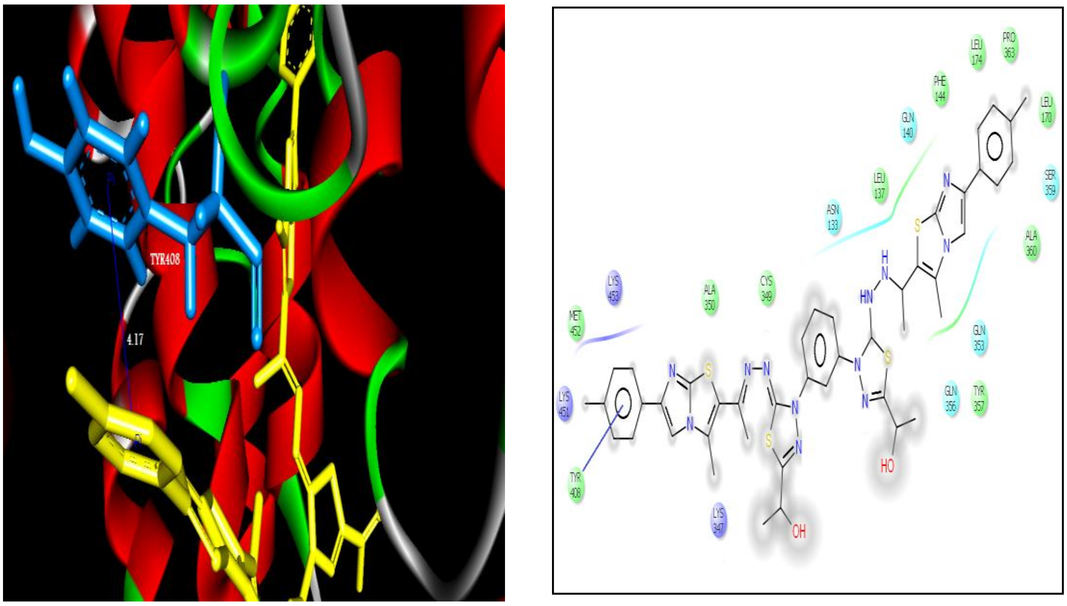

Compound **11**

Figure S1. Intermolecular interactions between the other docked molecules and GPC-3 protein. (Left) 3-Dimensional representation. (Right) 2- Dimensional representation.
